# Associations between Sjogren syndrome and psychiatric disorders in European populations: a 2-sample bidirectional Mendelian randomization study

**DOI:** 10.3389/fpsyt.2024.1465381

**Published:** 2024-10-16

**Authors:** Lingai Pan, Guangpeng Zhou, Guocui Wei, Qian Zhao, Yanping Wang, Qianlan Chen, Qing Xiao, Yujie Song, Xiangui Liang, Zhili Zou, Xiuxia Li, Xuan Xiong

**Affiliations:** ^1^ Department of Intensive Care Unit, Sichuan Provincial People’s Hospital, School of Medicine, University of Electronic Science and Technology of China, Chengdu, China; ^2^ Endocrine Department, Sichuan Provincial People’s Hospital, School of Medicine, University of Electronic Science and Technology of China, Chengdu, China; ^3^ Department of Pharmacy, Sichuan Provincial People’s Hospital, School of Medicine, University of Electronic Science and Technology of China, Chengdu, China; ^4^ Sichuan Provincial Center for Mental Health, Sichuan Provincial People’s Hospital, School of Medicine, University of Electronic Science and Technology of China, Chengdu, China

**Keywords:** Sjogren syndrome, psychiatric disorders, Mendelian randomization, genetic causal, bipolar disorder, schizophrenia

## Abstract

**Background:**

Psychiatric disorders, such as major depressive disorder (MDD), anxiety disorder (AD), bipolar disorder (BD), and schizophrenia (SCZ), are disturbances in brain activity that lead to disorders of cognition, behavior, and emotion regulation. Among Sjogren syndrome (SS) patients, psychiatric disorders are more prevalent than in the general population. Identifying associated risk factors can provide new evidence for clinical diagnosis and treatment.

**Methods:**

We selected genetic instruments based on published genome-wide association studies (GWASs) to determine predisposition. Then, we conducted a 2-sample bidirectional Mendelian randomization (MR) analysis to explore the potential causal associations between SS and four major psychiatric disorders. The primary analysis was performed using MR with the inverse-variance weighted method. Confirmation was achieved through Steiger filtering and testing to determine the causal direction. Sensitivity analyses were conducted using MR-Egger, MR-PRESSO, and “leave-one-out” method methods.

**Results:**

Our study showed that SS was linked to BD and SCZ, indicating that individuals with SS may have a reduced risk of developing BD (IVW: OR = 0.940, *P*=0.014) and SCZ (IVW: OR = 0.854, *P*=1.47*10-4), while there was no causal relationship between SS and MDD or AD. MR−Egger regression shows no evidence of pleiotropy (BD: intercept = 0.007, p = 0.774; SCZ: intercept = 0.051, p = 0.209). The same as the MR-PRESSO analysis (BD: global test p = 1.000; SCZ: global test p = 0.160). However, the results from the leave-one-out analysis demonstrated instability. Specifically, after excluding SNP rs3117581, the effects on BD and SCZ were found to be non-significant, suggesting the potential influence of unrecognized confounding factors. The results of the reverse MR show that four major psychiatric disorders had no causal effects on SS.

**Conclusions:**

Our research findings demonstrate a causal relationship between SS and SCZ, as well as between SS and BD. There are no causal effects between the four major psychiatric disorders and SS. These findings suggest that SS may have the potential to reduce the risk of both psychiatric disorders. This study provides new insight for their prevention and treatment.

## 1 Introduction

Psychiatric disorders are a group of intricate psychological syndromes, such as major depressive disorder (MDD), anxiety disorder (AD, such as generalized anxiety disorder, panic disorder, social phobia, specific phobias, and separation anxiety disorder), bipolar disorder (BD), and schizophrenia (SCZ), that affect cognitive, emotional, behavioral, and volitional health (1). Mental illness has been viewed as a significant burden on personal health care and the current healthcare system. Treatment and care are becoming increasingly challenging, and it is estimated that approximately 21.2%-32.4% of years lived with disability are due to psychiatric illness globally (2). Therefore, it is crucial to enhance our understanding of the pathophysiology and potential risk factors associated with psychiatric disorders to develop innovative strategies for prevention and intervention. The etiology of psychiatric disorders remains unclear. Emerging evidence suggests that the origin of psychiatric illness is complex and involves a combination of environmental and genetic influences, as well as parental psychopathological conditions (3).

Sjogren syndrome (SS) is a frequent systemic autoimmune disorder that predominantly affects the salivary and lacrimal glands, causing sicca complaints (4). It is estimated to be the second most common multisystem autoimmune disease after rheumatoid arthritis (5), with an incidence of approximately 0.22%-1.6% and a preponderance of middle-aged women (6). It also has a wide spectrum of clinical manifestations that essentially affect any organ system (7), including the musculoskeletal system (8). It is noteworthy that 2.7% to 9.8% of patients with SS may progress to non-Hodgkin lymphoma, with the risk increasing progressively with disease duration (9). Additionally, many patients with SS develop other rheumatic and autoimmune conditions, such as rheumatoid arthritis, autoimmune thyroid disease, primary biliary cholangitis, and systemic lupus erythematosus (10). These comorbidities further limit the patients’ social activities and functional abilities.

The impact of SS extends beyond individual patients, significantly affecting both societal and healthcare systems. The high prevalence of this disease necessitates increased allocation of medical and care resources for its diagnosis and treatment, posing a substantial challenge to public health systems.

Many studies have shown a strong correlation between mental illness and Sjogren’s syndrome (11–13). A study from Taiwan observed significantly increased incidences of MDD, AD, and sleep disorder in patients with SS (14). Eaton WW. et al. found that a history of any autoimmune disease was associated with a 45% increased risk of SCZ (15). The prevalence of nine autoimmune diseases was higher among SCZ patients compared to controls, with incidence rate ratios ranging from 1.9 to 12.5. Additionally, the prevalence of twelve autoimmune diseases was higher among the parents of SCZ patients compared to controls, with adjusted incidence rate ratios ranging from 1.3 to 3.8. Notably, the prevalence of SS was significantly higher in SCZ patients compared to the general population (RR = 3.5, 95% CI: 1.8-8.1, *P*<0.05). A meta-analysis showed that the incidence of depression in SS patients is estimated to be between 8.33% and 75.56% (16).

Rheumatoid arthritis (RA) is an autoimmune disease similar to SS. Current evidence indicates that the prevalence of RA is lower among patients with SCZ and their first-degree relatives, suggesting a potential protective effect of RA against SCZ (17).

The association between mental disorders and autoimmune diseases appears broader than previously suspected. Future studies on comorbidities may enhance the understanding of the pathogenesis of both mental disorders and autoimmune diseases. Given the limited data, there is a need for a better understanding of the causal association between SS and psychiatric disorders.

Mendelian randomization (MR) is an effective genetic epidemiological method used to assess the causal relationship between two phenotypes (18). By using genetic variants, such as single nucleotide polymorphisms (SNPs), as instrumental variables (IVs) to represent modifiable risk factors or exposures, MR design enhances causal inference in exposure–outcome associations (19). According to Mendel’s law of inheritance, genetic variations are randomly distributed during gamete formation, making gametes less susceptible to confounding factors (20). Furthermore, since the genotype remains stable throughout disease development, the influence of confounding factors and reverse causation can be minimized (21, 22).

To clarify the potential association between SS and psychiatric disorders, we conducted a two-sample bidirectional MR study using genome-wide association studies (GWASs). This research design allows for the examination of the impact of SS on the risk of psychiatric disorders. Clarifying these associations can provide valuable insights for clinical monitoring and contribute to the advancement of precision medicine in the future.

## 2 Methods

### 2.1 Study design

**Figure 1:**
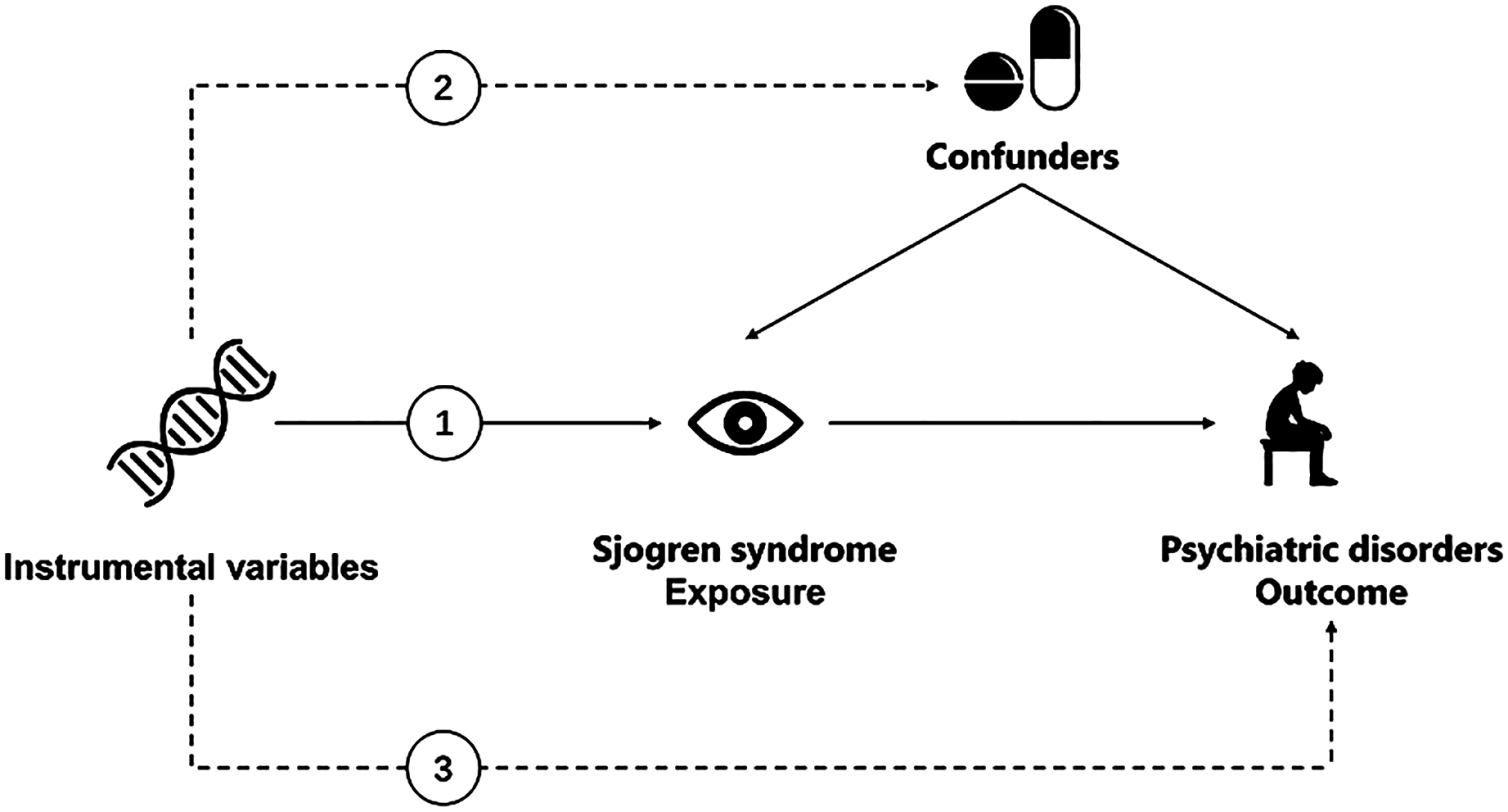
Overview of the Mendelian randomization framework used to investigate the associations between Sjogren Syndrome and psychiatric disorders. (1) The genetic variant is strongly correlated with exposure. (2) The genetic variant is independent of any potential confounders of the exposure-outcome association. (3) The genetic variant does not interact with the outcome independently of exposure.

The validity of MR analysis depends on three assumptions: (1) the genetic variant is strongly correlated with exposure, (2) the genetic variant is independent of any potential confounders of the exposure-outcome association, and (3) the genetic variant does not interact with the outcome independently of exposure (23–25). For this study, we performed a 2-sample bidirectional MR to examine the potential causal associations between SS and four psychiatric disorders (**Figure 1**) (26). First, we conducted a forward MR analysis to determine the positive causal relationship between SS and psychiatric disorders. Subsequently, we proceeded in the reverse direction to ascertain the causal relationship between the four psychiatric disorders and SS (27–30). Ethical approval and informed consent were not required because we used publicly available data sources.

### 2.2 Data sources

The relevant genetic variation associated with the four major psychiatric disorders was obtained from the meta-analysis of genome-wide association studies (GWAS) in the Integrative Epidemiology Unit (IEU) Open GWAS database (https://gwas.mrcieu.ac.uk/datasets). The respective sample sizes were as follows: MDD (59 851 cases and 113 154 controls), AD (Mental health problems ever diagnosed by a professional: Anxiety, nerves, or generalized anxiety disorder) with 16,730 cases and 101,021 controls, BD (20 352 cases and 31 358 controls), and SCZ (52 017 cases and 75 889 controls). The SS data were obtained from the FinnGen database (10th edition); the total sample size was 214435 patients (1290 cases and 213145 controls), and 16380454 SNPs were detected. To reduce the potential bias of population heterogeneity, genetic data from the European population dataset were used (31, 32). The corresponding GWAS IDs can be found in **Table 1**. More detailed information regarding the demographic characteristics of the summary data can be found in the original works (27–30).

**Table 1:** Characteristics of data in this study.

GWAS ID	Year	Trait	Ncase	Sex	Population	Sample size	Build	Ncontrol	Category	Nsnp	PMID
finn-b-M13_SJOGREN	2021	Sicca Syndrome	1290	Males and Females	European	214435	HG19/GRCh37	213145	Binary	16380454	NA
ieu-a-1188	2018	Major Depressive Disorder	59851	Males and Females	European	173005	HG19/GRCh37	113154	Disease	13554550	29700475
ukb-d-20544_15	2018	Anxiety Disorder	16730	Males and Females	European	117751	HG19/GRCh37	101021	Binary	13571547	NA
ieu-b-41	2019	Bipolar Disorder	20352	Males and Females	European	51710	HG19/GRCh37	31358	Binary	13413244	31043756
ieu-b-5102	2022	Schizophrenia	52017	Males and Females	European	127906	HG19/GRCh37	75889	Disease	NA	35396580

### 2.3 Selection of instrumental variables

For the selection of instrumental variables (IVs), P<5*10^-8^ was used as the threshold for extraction. If no significant SNPs were found at this threshold, an alternative threshold 5*10^−6^ used for extraction. Additionally, a linkage disequilibrium (LD) threshold of R^2^ < 0.001 and a clumping distance of 10,000 kb were applied to ensure that the instrumental variables were independent (33). In the second step, weak instrumental variables were excluded; that is, the F statistic was calculated as F=β ^2^/SE ^2^; SNPs with F <10 were excluded (34). In the third step, we further analyzed whether the SNPs were associated with confounding factors using the PhenoScanner database. This study only included SNPs that were not associated with confounding factors, while including instrumental variables related to SS. Steiger filtering was used to test the directionality of the association of the remaining instrumental, variables with the outcome. Any instrumental variable labeled as FALSE by Steiger filtering, indicating that the SNP explained more variance in the outcome than in the exposure, was excluded from the MR analysis. Finally, the SNPs associated with the confounding factors were deleted, and appropriate SNPs were retained so that the effect values of exposure and outcome corresponded to the same effect allele. After harmonizing the exposure and outcome data, palindromic SNPs with intermediate allele frequencies were removed (35).

### 2.4 MR analyses

MR analysis was conducted using R software (version 4.0.3) along with the R packages “TwoSampleMR”, “MR_PRESSO”, and “coloc”. In our MR analysis, we utilized the inverse-variance weighted (IVW), weighted median method (WME), MR-Egger regression, and outlier (MR-PRESSO) methods, which are four distinct methods used to evaluate the associations between SS and the four psychiatric disorders (36–39). The characteristic of IVW is that it does not consider the intercept term and aligns with the inverse variance of the instrumental variable as the weight. To ensure that all instrumental variables are valid and that there is no pleiotropy, the SNPs are calculated one by one using the ratio method, and weighted regression is performed to obtain the overall estimated value (40). The WME requires effective instrumental variables to exceed 50% for weighted weight calculation (41). By arranging SNPs by weight size and taking the median as the result, this method consistently produces consistent causal estimates (42). The MR-Egger method differs from the IVW method in that it considers the presence of intercept terms and uses the reciprocal of the outcome variance as the weight for fitting (43). This study focuses on the IVW results, and only when the IVW results are statistically significant and the effect values of the four methods are in the same direction will positive results be considered.

### 2.5 Sensitivity analyses

In our study, Cochran’s Q test was used to test for heterogeneity, and a funnel plot was utilized for the IVs (44). When *P*>0.05, the results showed that there was no heterogeneity. If the p value was less than 0.05, the IVW random effects model was chosen to eliminate heterogeneity. Horizontal pleiotropy is very important to our study because the effect estimation may be unstable under the influence of horizontal pleiotropy. The horizontal pleiotropy test was performed mainly by using the MR−Egger intercept (45). The MR−Egger intercept method was used to estimate the possibility of horizontal pleiotropy by calculating the intercept term that could be obtained after linear regression analysis (46). *P*>0.05 indicated that the results had no pleiotropy. Furthermore, the “leave-one-out” method was used to analyze the robustness of the results (47). The symmetry of the funnel plot was visually inspected to assess whether the SNPs included in the analysis had outliers. Through these comprehensive analyses, we considered the IVW (fixed/random effects) method as the primary estimator for causal effects and assessed the consistency across all MR methods. Furthermore, we conducted a colocalization analysis using the commonly used Bayesian model to investigate whether SS and psychiatric disorders share a common causal variant in a given region (48). For the SS gene locus where there was evidence supporting a causal relationship with psychiatric disorders (*P* < 0.05), variables within 200 kb of the corresponding instrumental SNP were extracted and sent to calculate the posterior probability (PP) (49). As a convention, a PP.H4 of 0.75 or higher was considered evidence of colocalization (50). For each obtained exposure-outcome pair, the study further tested the direction of the causal relationship through Steiger directionality test to avoid reverse causation.

## 3 Results

### 3.1 Causal effects of SS on the risk of psychiatric disorders

**Figure 2:**
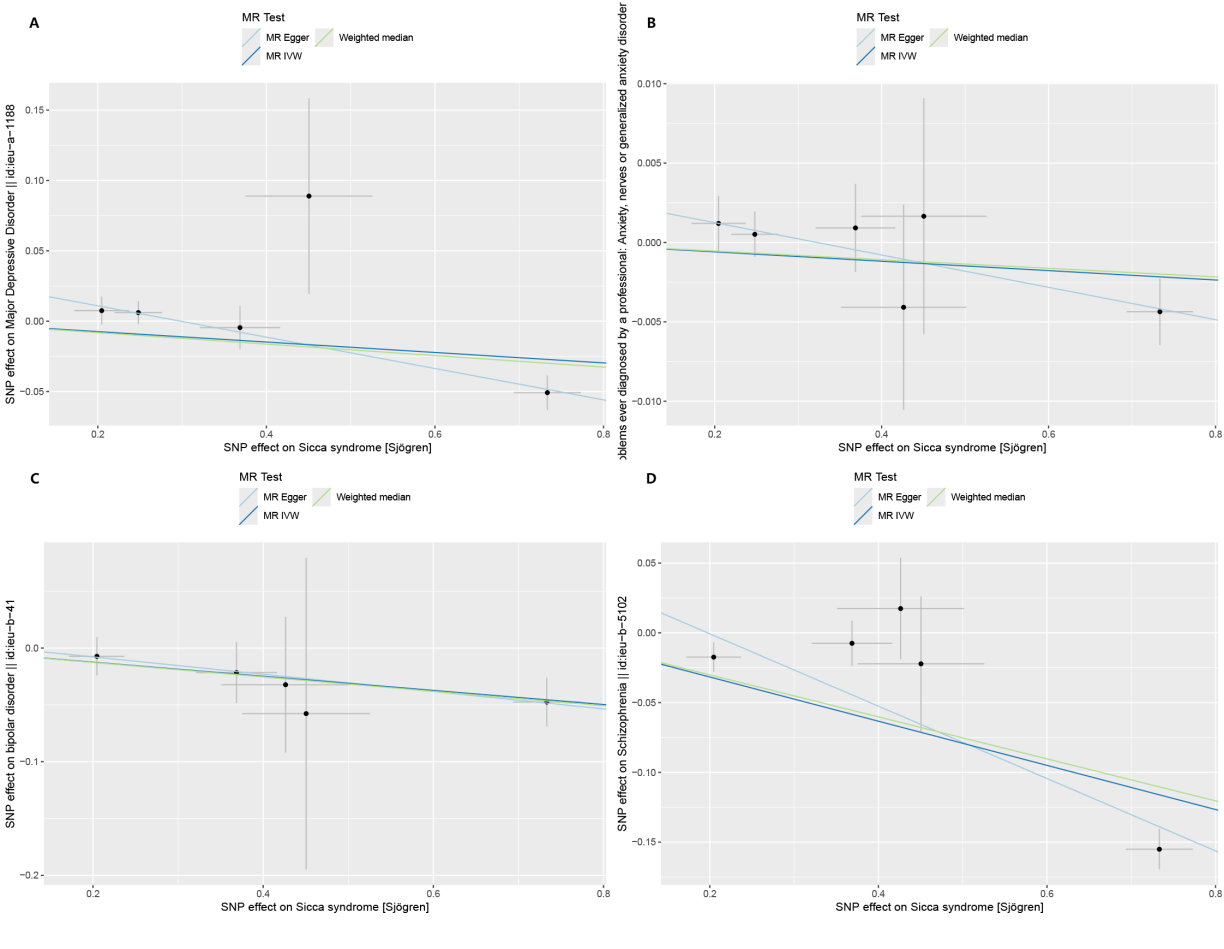
Scatter plots of Mendelian randomization regressions for Sjogren syndrome (exposure) and psychiatric disorders (outcome). **(A)** Major depressive disorder (MDD). **(B)** Anxiety disorder (AD). **(C)** Bipolar disorder (BD). **(D)** Schizophrenia (SCZ). MR, Mendelian randomization; IVW, inverse-variance weighted standard Mendelian randomization analysis; MR-Egger, Mendelian randomization-Egger pleiotropy-adjusted regression.

**Figure 3:**
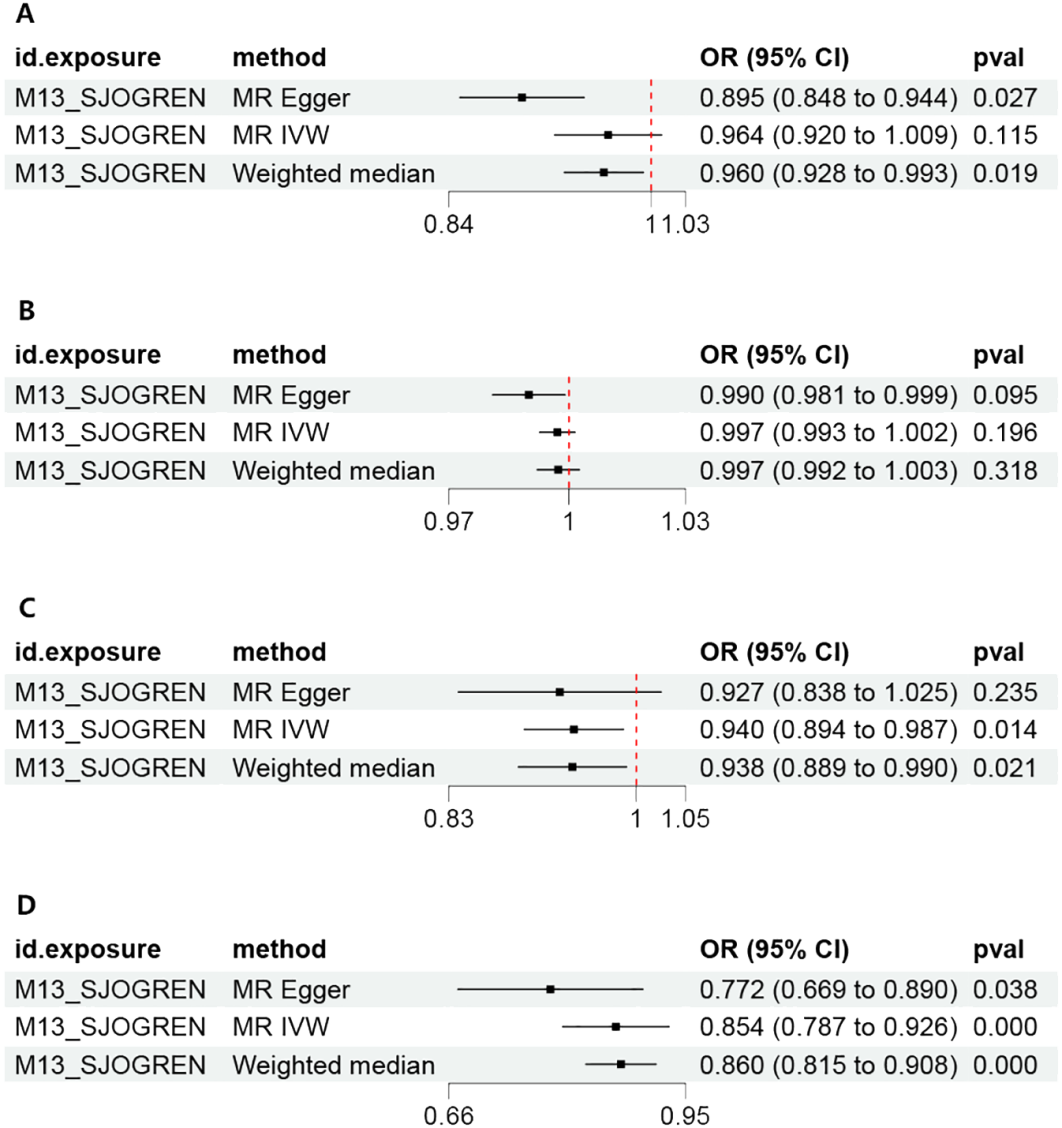
Forest plot of the MR estimates of Sjogren syndrome on psychiatric disorders. **(A)** Major depressive disorder (MDD). **(B)** Anxiety disorder (AD). **(C)** Bipolar disorder (BD). **(D)** Schizophrenia (SCZ). MR, Mendelian randomization; IVW, inverse-variance weighted standard Mendelian randomization analysis; MR-Egger, Mendelian randomization-Egger pleiotropy-adjusted regression; OR, Odd Ratio.

**Figure 4:**
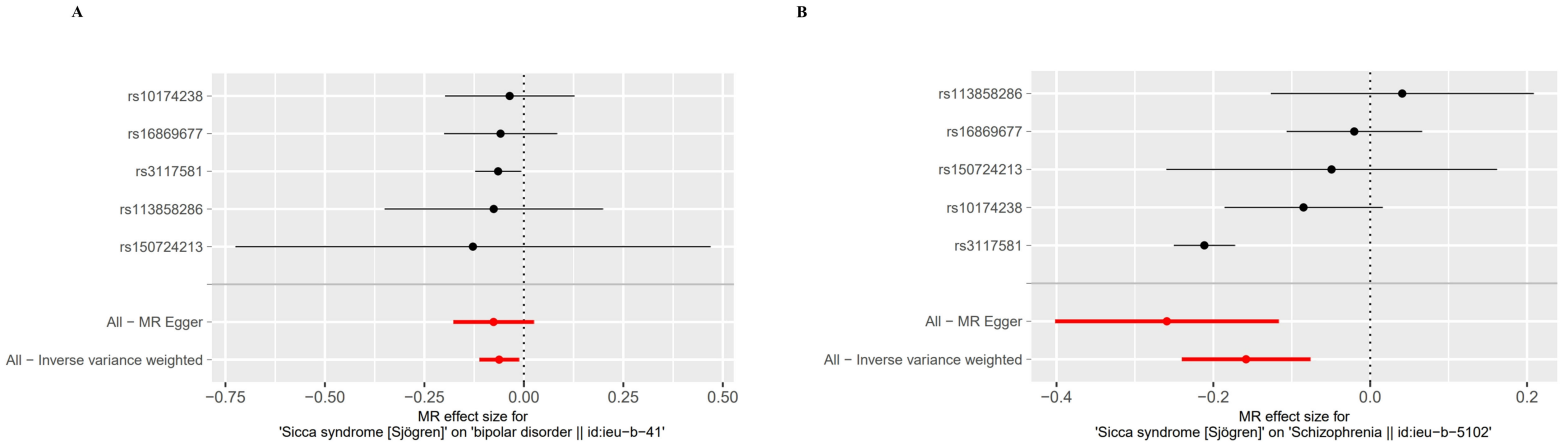
Forest plot of the MR Effect size for Sjogren syndrome on psychiatric disorders. **(A)** Bipolar disorder (BD). **(B)** Schizophrenia (SCZ).

By setting a correlation threshold with the exposure, and performing linkage disequilibrium removal, 7 SNPs from SS were obtained (**Supplementary Table 1**). The F-values ranged from 32.17 to 340.20 (**Supplementary Table 2**). Phenoscanner analysis did not identify any confounding SNPs (**Supplementary Table 3**). No instrumental variables were flagged as FALSE by Steiger filtering (**Supplementary Table 4**). Additionally, steiger directionality testing was also used to confirm the directional accuracy of the associations between SS with BD and SCZ traits (**Supplementary Table 5**). Therefore, evaluations of weak instruments, confounding factors, and Steiger filtering did not exclude any SNPs from the MR analysis. After aligning exposure and outcome data and excluding palindromic SNPs with intermediate allele frequencies (MDD: rs9272305, rs113858286; AD: rs9272305), the following SNPs were identified as genetic instrumental variables: 5 for MDD, 6 for AD, 5 for BD, and 5 for SCZ. MR analysis was carried out using four methods. We compared the four MR analyses of major psychiatric disorders: MR Egger, the weighted median, and MR_PRESSO produced directionally consistent effects as the IVW estimates. The MR analysis results are presented as odds ratios (ORs) for psychiatric disorders per standard deviation (SD) increase or decrease in SS. The results of the MR analysis are presented in **Figure 2**, while the forest plot demonstrates that both the MR-Egger method and the IVW method confirm a causal effect of SS on BD and SCZ (**Figure 3**). We found evidence that higher genetically predicted SS was associated with a decreased risk of BD (IVW: OR = 0.940, 95% CI =0.894–0.987, *P*=0.014) and SCZ (IVW: OR = 0.854, 95% CI =0.787–0.926, *P*=1.47*10^-4^) but was not associated with the other two psychiatric disorders. To control the false positive discovery rate, we adjusted the p-values using the Benjamini-Hochberg method. After adjustment, the association between SS and BD (FDR= 0.027), as well as between SS and SCZ (FDR= 5.88784*10^-4^), remained significant. The relationships between SS and the risk of the four psychiatric disorders are shown in **Table 2**. The SNPs effect size for SS on BD and SS on SCZ in **Figure 4**.

**Table 2:** Summary of the MR-based analysis of Sjogren syndrome and psychiatric disorders.

Exposure	Outcome	SNPs	IVW				Weighted median			MR-Egger			MR_presso			Heterogeneity tests	Horizontal pleiotropy	
			β	OR	P	β	FDR	OR	P	β	OR	P	β(Causal Estimate)	P	Global_Test_Pvalue	Q_pval	Egger_intercept	pval
SS	MDD	5	-0.03707	0.96361	0.114535	-0.04075	0.152713	0.960066	0.018722	-0.1114	0.89458	0.026523	-0.031022804	0.19961	0.11	0.015376097	0.033203025	0.052111404
SS	AD	6	-0.00295	0.997052	0.196208	-0.00271	0.196208	0.997293	0.31776	-0.01018	0.989867	0.094794	-0.002198234	0.45252	0.38	0.576868383	0.003286394	0.150701749
SS	BD	5	-0.06211	0.93978	0.01371	-0.06352	0.027419	0.938455	0.020526	-0.07618	0.926646	0.235154	-0.062109743	0.00028	1	0.996602969	0.007431332	0.774171184
SS	SCZ	5	-0.15831	0.853581	0.000147	-0.15056	0.000589	0.860229	4.23E-08	-0.2593	0.771594	0.037551	-0.158314433	0.01917	0.16	3.80972E-05	0.051156573	0.209419129
MDD	SS	4	-0.12535	0.88219	0.896335	0.281523	0.896335	1.325147	0.645391	-9.17849	0.000103	0.091766	-0.020511387	0.97849	0.03	0.004044411	0.452004249	0.091761023
AD	SS	15	-2.46735	0.084809	0.092061	-1.85563	0.368243	0.156354	0.367657	-0.21904	0.803294	0.948252	-2.467347851	0.05826	0.78	0.80847222	-0.028682513	0.462357304
BD	SS	13	0.144618	1.155598	0.327469	0.262331	0.654939	1.299956	0.188856	-0.48784	0.613952	0.599242	0.178480292	0.15265	0.7	0.53221018	0.058997027	0.49183805
SCZ	SS	144	0.018608	1.018782	0.755335	0.095373	0.896335	1.100069	0.270461	-0.39197	0.675724	0.113243	0.014454601	0.80467	0.285714286	0.304427557	0.027694687	0.087603562

SNP, single nucleotide polymorphism; SS, Sjogren syndrome; MDD, Major depressive disorder; AD, Anxiety disorder; BD, Bipolar disorder; SCZ, Schizophrenia; IVW, inverse variance weighting; MR-Egger, Mendelian randomization-Egger pleiotropy-adjusted regression.

### 3.2 Causal effects of psychiatric disorders on the risk of SS

MR analyses of four major psychiatric disorders were conducted for the 2-sample MR analyses of the SS. Since no significant SNPs were found at the5*10^−8^ threshold, we used the 5*10^−6^ threshold for AD to SS. The SNPs (MDD: 4, AD:15, BD: 13, SCZ: 144) applied as genetic IVs for psychiatric disorders after excluding palindromic SNPs (MDD: rs76025409; AD: rs17164793, rs1694895; BD: rs5758065, rs10455979, rs2314398; SCZ: rs61857878, rs13011472, rs1540840, rs1892346, rs1914399, rs1953205, rs3770754, rs4700418) are shown in **Supplementary Table 6**. To ensure that no weak IVs were included after the threshold changed, we also calculated F values, and there were no SNPs with F <10 (**Supplementary Table 7**). The results of Phenoscanner analysis are shown in **Supplementary Table 8**. The results showed that four major psychiatric disorders had no causal effects on SS (**Table 2**).

### 3.3 Sensitivity analyses did not reveal any indication of unknown pleiotropy.

**Figure 5:**
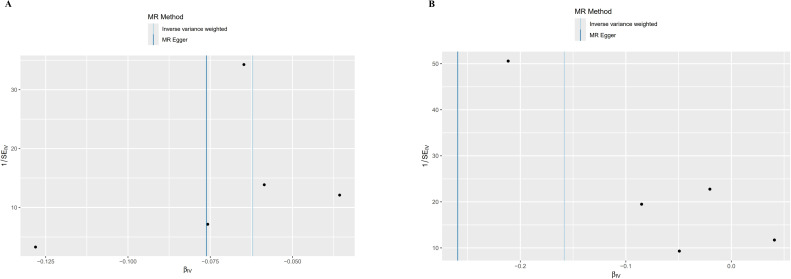
Funnel plot of MR analysis results. SE, Standard Error; β, Effect Size; MR Method, Mendelian Randomization Method; Inverse variance weighted: Inverse Variance Weighted Method; IV, Instrumental Variable. **(A)** Bipolar disorder (BD). **(B)** Schizophrenia (SCZ).

**Figure 6:**
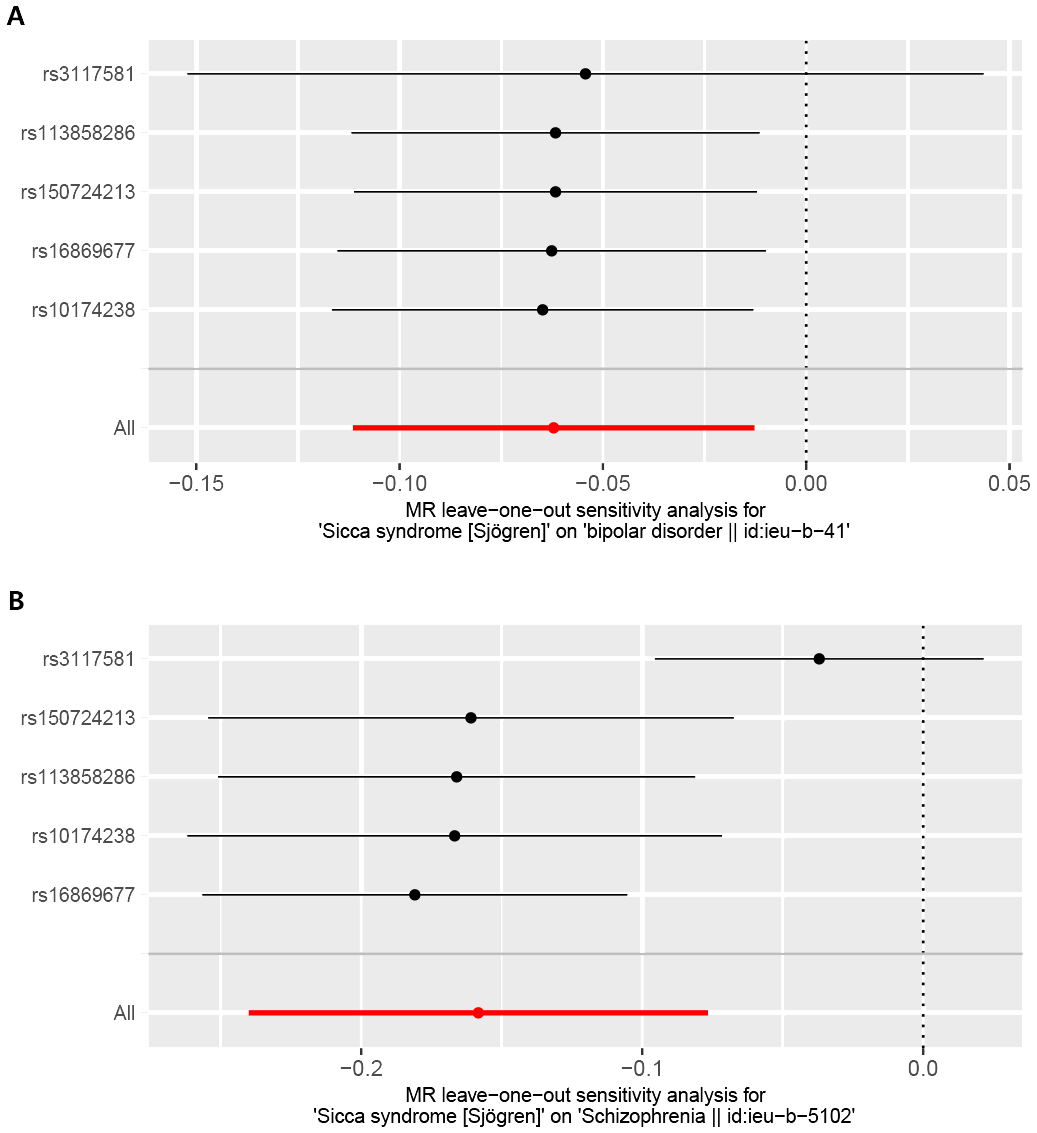
Leave-one-out plot for the causal association between Sjogren syndrome (exposure) and psychiatric disorders (outcome) after omitting each SNP. **(A)** Bipolar disorder (BD). **(B)** Schizophrenia (SCZ).

The foundation of Mendelian randomization studies for determining causality is the absence of pleiotropic biases. To assess potential biases, we used the PhenoScanner database to examine the biological pleiotropy of the SNPs involved. Each included SNP was individually examined for the associated phenotype. The sensitivity analyses revealed heterogeneity in the Cochran’s Q test (Q-p=3.81*10^-5^) of SCZ patients, which the funnel plot suggested the same (**Figure 5A**). Therefore, the IVW random effects model was selected. Furthermore, the Cochran’s test (Q-p=0.997) and funnel plot of BD showed no evidence of heterogeneity (**Figure 5B**). The MR-PRESSO global test (BD: global test p=1.000, SCZ: global test p=0.160) and MR−Egger intercept (intercept = 0.007, p = 0.774; SCZ: intercept = 0.051, p = 0.209) did not reveal any evidence of horizontal pleiotropy (**Table 2**). The black dots in the leave-one-out sensitivity analysis figure represent the estimated causal associations between each specific exposure and the target mental disorder when each SNP is removed one at a time. The red dots indicate the overall causal estimate using the random-effects inverse variance weighted method, while the horizontal lines denote the 95% confidence intervals. The results from the leave-one-out analysis show that some rsID positions exceed the line of the null (no association). After excluding top SNPs rs3117581, the effects show non-significant for both BD and SCZ (**Figure 6**). Additionally, in our study, the results of the colocalization analysis shown in **Supplementary Table 9** indicate that the probability of the H4 hypothesis being less than 75% suggests that the causal relationship between SS and BD or SS and SCZ is not driven by the same SNP within their genetic sequences (**Supplementary Figure 1**).

## 4 Discussion

Numerous clinical cases and controlled studies have demonstrated that the incidence of mental illness in patients with Sjögren’s syndrome exceeds that observed in the general population; however, the precise cause remains unknown (51). Our study, based on the Diagnostic and Statistical Manual of Mental Disorders, Fifth Edition (DSM-5), selected the most common psychiatric disorders from four disease spectra as research subjects (52). To the best of our knowledge, this is the first MR analysis study to determine the correlation between Sjogren’s syndrome and psychiatric disorders from a genetic perspective.

SS is a systemic autoimmune disease characterized by immune hyperfunction, lymphocyte proliferation, progressive destruction of exocrine glands, and the presence of autoantibodies (53, 54). The common clinical signs of SS include dryness, fatigue, and muscle pain, as well as systemic symptoms (55). Currently, the main diagnostic criteria are based on the 2002 SS International Classification Diagnostic Standards or the 2016 American College of Rheumatology/European Alliance of Associations for Rheumatology (ACR/EULAR) diagnostic standards, with a sensitivity and specificity of 96% and 95%, respectively (56). The onset of SS is implicit, with clinical manifestations involving multiple systems and requiring multidisciplinary team (MDT) management.

Many studies have reported a high rate of anxiety and depression in SS patients (57–59). In a study conducted in the UK, it was found that the average scores on depression and anxiety scales for the SS group were significantly higher than those of the control group. Additionally, the prevalence of depression in the SS group was reported to be 15% (60). Cui et al. (61) reported that anxiety was responsible for 33.8% and depression was responsible for 36.9% of Chinese SS patients, which was significantly greater than that in the control group. Studies have shown that in individuals with SS, depression and anxiety disorders occur, with female depression being the main disorder and male anxiety being more common (62). The risk of depression is highest among individuals aged 65 to 80 years (63). A large study reported that the most common mental disorder in SS patients is depression, and the risk of being newly diagnosed significantly increases in the first and fifth years after diagnosis (64). Research in Türkiye showed that 31.1% of SS patients suffer from depression, and 30% suffer from anxiety (65). The quality-of-life scores for patients with anxiety or depression were significantly lower than those of the control group (66). A meta-analysis revealed that depression rates (OR=2. 65, 95% CI (2. 07, 3. 38), P<0. 001) and anxiety (OR=2. 19, 95% CI (1. 86,2. 57), P<0. 001), are significantly different in SS patients than in the general population (16).

Research has shown that in mammals, the neurotransmitter 5-HT, which is closely related to anxiety and depression, is produced by tryptophan through the 5-HT pathway (67–70). Tryptophan has two different metabolic pathways, the kynurenine pathway (KP), in which 90% of tryptophan is metabolized through KP (71). Indoleamine 2,3-dioxygenase (IDO) is the first rate-limiting enzyme in the KP pathway, catalyzing the production of canine uric acid from the substrate tryptophan; when IDO increases, it further increases tryptophan metabolism through the KP, thereby affecting the production of 5-HT (72, 73). IDO is an inflammation-inducible enzyme that can be activated by various inflammatory factors, including interferon (IFN)-γ and tumor necrosis factor (TNF)-α (74). Under cytokine stimulation, IDO is upregulated in patients with depression, which abnormally activates the KP metabolic pathway, interferes with the transmission of serotonin and glutamate neurotransmitters, and induces neurological manifestations, leading to pain hypersensitivity and depressive symptoms (75). Clinical studies have shown that the levels of IL-1, IL-6, TNF-α, and IFN-α are generally elevated in SS patients; therefore, some people believe that SS is a cause of depression and anxiety (76). Unlike clinical research results, our study revealed no causal relationship between ss and depression and anxiety from a genetic perspective, maybe elevated IL-1, IL-6, TNF-α and IFN-α levels are not related to the pathogenesis of SS. This is consistent with the research findings that drugs such as IL-1 antagonists and TNF-α antagonists cannot significantly improve the condition of SS (77–79). The depression hypothesis suggests that the frontal lobe, temporal lobe, thalamus, and other areas are related to depression-related neural circuits, causing damage to neurons and nerve fibers in these areas, as well as impaired neurotransmitter receptor function, which is a significant cause of depression (80). Therefore, the clinical relevance may be due to cerebral vascular injury caused by SS immune inflammation, which is related to emotionally related cerebral ischemia and bleeding (81). In addition, the impact of medication may also contribute to the increased prevalence of anxiety and depression in SS patients. SS is a chronic condition characterized by diffuse connective tissue disease. The side effects of long-term use of hormones and immunosuppressants, such as hormonal imbalances, menstrual disorders, and changes in appearance and body shape, also cause anxiety and depression. Huang et al. conducted a study on predictive factors for depression in patients with systemic lupus erythematosus, and the results showed that depression in patients with SLE is multifactorial, high-dose prednisone (≥20 mg/d) being an important independent risk factor (82). This suggests that long-term routine SS treatment may be correlated with the development of anxiety and depression.

Neuroinflammation has been increasingly recognized as a significant factor contributing to the pathophysiology of various mental health conditions. Evidence suggests that neuroinflammation can disrupt neurochemical balance, impair neuroplasticity, and alter neuronal function, thereby exacerbating psychiatric symptoms and potentially initiating or worsening mental illnesses (83). Furthermore, the impact of neuroinflammation extends beyond psychiatric disorders, influencing other neurodegenerative diseases as well. Chronic neuroinflammation is implicated in the progression of conditions such as Alzheimer’s disease, Parkinson’s disease, and multiple sclerosis (84). In these diseases, persistent inflammation contributes to neuronal damage, cognitive decline, and motor dysfunction.

Currently, increasing evidence suggests a connection between SCZ and neuroinflammation, particularly regarding the cytokines involved in inflammatory responses (85). Research shows that maternal exposure to Toxoplasma gondii and measles viruses during pregnancy can increase the risk of offspring developing SCZ (86). The clearance of pathogens such as Toxoplasma gondii and measles virus is mediated by Th1 cell immune responses (87). IL-12 serves as the most critical inducer for Th1 cell differentiation, promoting the maturation of Th1 cells (88). SS is considered an autoimmune disease mediated by Th1-type immune responses. In patients with SS, abnormal production of IL-12 occurs in monocytes, macrophages, and dendritic cells, leading to elevated plasma IL-12 levels, which are associated with disease activity (89). This indicates that IL-12 is involved in the pathogenesis of SS. The increased IL-12 levels in SS patients induce Th1 cell differentiation, resulting in the aberrant activation of Th1-type immune responses, which enhances pathogen clearance and potentially reduces the risk of schizophrenia (SCZ). This may represent a genetic mechanism underlying the relationship between SS and SCZ.

Studies have shown that an increase in CRP is negatively correlated with the occurrence of SCZ, indicating that an increase in CPR can reduce the likelihood of SCZ (90). Observational studies have shown that low levels of certain acute phase proteins in newborns are associated with a greater risk of SCZ, and compared to newborns in the control group, newborns who develop SCZ later have an impaired ability to increase acute phase protein levels (such as CRP) in response to certain maternal infections (91). In adults, prospective studies have shown that higher CRP levels are associated with increased susceptibility to infection (92, 93). This suggests that in the early stages of the disease, the immune defense system, including CRP, is upregulated via negative feedback to resist external invasion and delay disease progression. Due to the hyperactive immune function in patients with SS, their CRP levels are typically higher than those of the normal population. This may play an anti-inflammatory role, thereby reducing the risk of SCZ.

Additionally, our study identified that when SS was considered as an exposure, the selected instrumental variables rs10174238 and rs2004640, which are associated with RA, were located in the STAT4 and IRF5 genes, respectively. Numerous studies have demonstrated a negative correlation between SCZ and RA (94–96). Therefore, the protective effect of SS on SCZ may be attributed to rs10174238 and/or rs2004640.

While many observational studies have reported an increased risk of SS in patients with SCZ (15), our MR analysis finds no harmful causal effect of SCZ on SS but rather a protective effect. This discrepancy may arise because correlations observed in observational studies do not necessarily imply causation. Various confounding factors, such as comorbidities, socioeconomic conditions, and lifestyle choices, could influence the increased risk of SS seen in SCZ patients.

SCZ and BD have similar pathological features, with damage to the dendritic spines on the dorsolateral side of the prefrontal cortex and a decrease in gray matter in the emotional processing areas (anterior cingulate gyrus and anterior cingulate gyrus) (97). Compared to healthy controls (HCs), SCZ and BD patients exhibit changes in brain morphology, including impaired white matter (WM) connectivity (98, 99). Furthermore, SCZ and BD exhibit significant overlap in genetic risk factors (100), as well as consistency in clinical features (101), neuropsychological disorders (102), ventricular enlargement, and overall brain volume reduction (103). Our study also revealed that genetic characteristics, such as SS, reduce the likelihood of two diseases from a genetic perspective.

## 5 Limitations

Despite rigorous selection of instrumental variables in this study—including the exclusion of weak instruments, palindrome structure SNPs, and SNPs associated with confounding factors—several limitations remain. Specifically, in the reverse Mendelian randomization analysis, the initially set threshold failed to identify AD-related SNPs, necessitating an adjustment to the threshold. This adjustment may introduce potential bias. Statistical results indicate that the leave-one-out analysis in the forward Mendelian randomization study exhibited instability, suggesting the possible presence of unknown confounding factors.

Overall, the interpretation of this study’s results should be approached with caution. Future research, particularly large-scale clinical or epidemiological studies, is recommended to validate these findings. While the GWAS data utilized represents a substantial sample size, limitations persist. Given the current lack of GWAS data for SS and PD in other populations, this study is confined to the European cohort. Future research should aim to incorporate GWAS data from other populations to complement and enhance the findings of this study.

## 6 Conclusion

In conclusion, our MR analysis revealed a protective causal effect of SS on the risk of SCZ and BD. This finding suggests that the positive associations of SS with psychiatric disorder risk in conventional observational studies may be due to limitations such as reverse causation or residual confounding. Although our findings may be due to horizontal pleiotropy that we failed to detect and account for, they at least suggest that SS does not significantly increase the risk of psychiatric disorders. This association may be related to the immune system hyperactivity mechanism caused by SS, providing new evidence for the anti-inflammatory treatment pathways of BD and SCZ and confirming that anti-inflammatory drugs have deeper research value in improving BD and SCZ.

## Data Availability

The original contributions presented in the study are included in the article/**Supplementary Material**. Further inquiries can be directed to the corresponding author.
