# Supplementary figures and images for: Associations between Sjogren syndrome and psychiatric disorders in European populations: a 2-sample bidirectional Mendelian randomization study
Source: Front Psychiatry. 2024 Oct 16;15:1465381. doi: 10.3389/fpsyt.2024.1465381 (PMC11521899; doi:10.3389/fpsyt.2024.1465381)

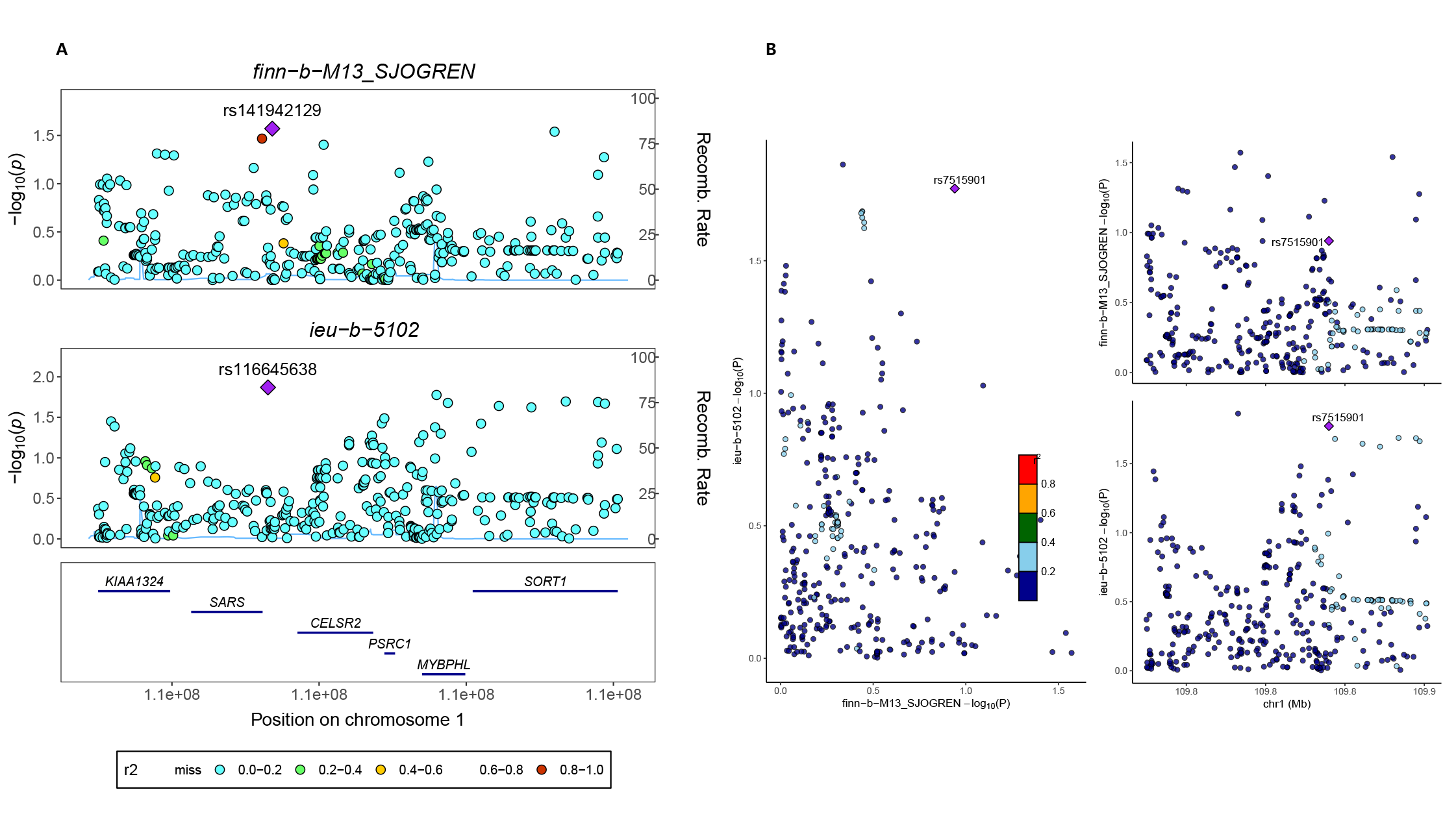

Supplement: Supplementary file 1 [file DataSheet1.zip › Figure S1.TIF]
